# A pan-cancer assessment of alterations of the kinase domain of ULK1, an upstream regulator of autophagy

**DOI:** 10.1038/s41598-020-71527-4

**Published:** 2020-09-10

**Authors:** Mukesh Kumar, Elena Papaleo

**Affiliations:** 1 Computational Biology Laboratory, Center for Autophagy, Recycling and Disease (CARD), Danish Cancer Society Research Center, Strandboulevarden 49, 2100 Copenhagen, Denmark; 2 Translational Disease System Biology, Faculty of Health and Medical Sciences, Novo Nordisk Foundation Center for Protein Research, University of Copenhagen, Copenhagen, Denmark

**Keywords:** Computational biology and bioinformatics, Molecular modelling

## Abstract

Autophagy is a key clearance process to recycle damaged cellular components. One important upstream regulator of autophagy is ULK1 kinase. Several three-dimensional structures of the ULK1 catalytic domain are available, but a comprehensive study, including molecular dynamics, is missing. Also, an exhaustive description of ULK1 alterations found in cancer samples is presently lacking. We here applied a framework which links -omics data to structural protein ensembles to study ULK1 alterations from genomics data available for more than 30 cancer types. We predicted the effects of mutations on ULK1 function and structural stability, accounting for protein dynamics, and the different layers of changes that a mutation can induce in a protein at the functional and structural level. ULK1 is down-regulated in gynecological tumors. In other cancer types, ULK2 could compensate for ULK1 downregulation and, in the majority of the cases, no marked changes in expression have been found. 36 missense mutations of ULK1, not limited to the catalytic domain, are co-occurring with mutations in a large number of ULK1 interactors or substrates, suggesting a pronounced effect of the upstream steps of autophagy in many cancer types. Moreover, our results pinpoint that more than 50% of the mutations in the kinase domain of ULK1, here investigated, are predicted to affect protein stability. Three mutations (S184F, D102N, and A28V) are predicted with only impact on kinase activity, either modifying the functional dynamics or the capability to exert effects from distal sites to the functional and catalytic regions. The framework here applied could be extended to other protein targets to aid the classification of missense mutations from cancer genomics studies, as well as to prioritize variants for experimental validation, or to select the appropriate biological readouts for experiments.

## Introduction

Autophagy is a highly conserved catabolic mechanism across eukaryotes to degrade different cellular components and molecules, including organelles, proteins, and bacteria^1,2^. Autophagy initiates with the formation of the autophagosome, which later fuses to the lysosome, resulting in the degradation of the cargo and the release of cellular building blocks^3,4^. At a basal level, autophagy contributes maintaining cellular homeostasis and the recycling of cellular components. Autophagy is also induced as a response to different stresses with a cytoprotective role. Defects in the autophagy machinery are often linked to diseases, including cancer, neurodegeneration, and bacterial infections^5^.

The ULK1 (Unc-51 like autophagy activating kinase 1) complex initiates autophagy^6–8^. The complex consists of ULK1kinase, FIP200 (FAK family kinase interacting protein of 200 kDA) scaffold protein, and the ATG13-ATG101 HORMA (Hop/Rev7/Mad2) complex^9^. The ULK1 complex can integrate different signals to promote both bulk and selective autophagy^9^. A highly coordinated and conserved cascade of post-translational events of the ULK1 complex, including phosphorylation and ubiquitination acts as major switch for autophagy initiation^10–13^. In most of the cases, autophagy initiation is regulated by the interplay between mTOR (mammalian Target of Rapamycin) and AMPK (AMP-activated protein kinase) complexes. These complexes perform a series of inhibitory or activatory phosphorylations of the ULK1 complex in different physiological conditions^10,14–18^. Upon activation, ULK1 directly phosphorylates the PI3K kinase complex, including BECLIN-1, VPS34, and ATG14, which, in turn, facilitate nucleation of the phagophore membrane at the phagophore assembly sites^19–21^**.** ULK1 also phosphorylated AMBRA1, which is a positive regulator of the PI3K complex^21^**.** By analogy to the yeast counterpart, ULK1 has been suggested to play essential scaffolding roles for autophagosome formation and maturation^9^. Mammals have five ULK1 homologs with ULK1 and ULK2 featuring the highest similarities and functional redundancy^7^, implying that both need to be inactivated for a substantial inhibition of autophagy^22^.

Different cancer types or subtypes show a deregulation of ULK1^23–26^. Autophagy, in general, has a strong association with cancer and can act with a dual role in a context-dependent way, being both tumor suppressor or promoter^27,28^. AMPK-ULK1 mediated autophagy induces resistance against bromodomain and extraterminal domain inhibitors, which are novel epigenetic therapeutics for acute myeloid leukemia^29^. Several inhibitors of ULK1 have been used to study its function in autophagy^21,30–32^. These molecules have potential to be used in cancer therapy in light of cancer addiction to autophagy^33^. Moreover, ULK1 is involved in the first biochemical steps of autophagy, representing an amenable druggable target in the pathway. X-ray structures of ULK1 in complex with inhibitors are available (PDB entries: 4WNO^31^, 4WNP^31^, 5CI7^32^, 6QAS^34^, and 6MNH^35^).

ULK1 is a multi-domain serine-threonine kinase, enriched in disordered regions^36^, which is a common trait of many scaffolding proteins. ULK1 has preference for serine as the phospho-acceptor residue in the substrate and hydrophobic residues surrounding the phosphorylation site^21^. The kinase domain is located at the N-terminal region of the protein and conserved among yeast and mammals^9^. The N-terminal kinase domain of ULK1 includes the activation loops (165–174 and 178–191) and the catalytic loop (136–145). ULK1 also includes a proline/serine-rich region (279–828) and a C-terminal domain (829–1,051), which are involved in interactions with ATG13, FIP200, and other upstream regulators such as mTOR and AMPK^15,37^. On the contrary, the N-terminal kinase domain of ULK1 could interact with the LRKK2 protein^38^. An important activatory autophosphorylation site is located in the activation loop at T180^9^ which, upon phosphorylation, can engage in salt bridges with the neighboring arginine residues R137 and R170^31^.

Despite the availability of X-ray structures of ULK1 kinase domain, no extensive molecular dynamics (MD) simulation studies of the protein have been undertaken. The availability of a conformational ensemble of a protein is of paramount importance to better understand its function and the effects of its alterations due to mutations^39–42^. Thus, we used all-atom and coarse-grain models to obtain an ensemble of conformations and account for ULK1 flexibility and dynamics. We then applied methods inspired by network theory to achieve a Protein Structure Network representation^43,44^ of the conformational ensemble. This methodology can help to identify residues important for structural stability and function, along with to pinpoint possible and elusive effects triggered by distal sites with respect to the functional residues of the protein^45–47^.

We combined these structural studies with a curation of alterations of ULK1 in more than 30 cancer studies available in The Cancer Genome Atlas (TCGA)^48,49^, with attention to changes in expression level or due to mutational events of the protein itself. Indeed, TCGA is among the most important examples of large-scale cancer genomics studies, collecting clinical and molecular data for over 33 tumor types and more than 11,000 samples from cancer patients. Both expression and mutational data are available for TCGA samples. In addition, since the pool of normal samples is often underrepresented or not available in TCGA, the Recount and Recount2 initiatives^50,51^ aimed at integrating TCGA data with normal healthy samples for the Genotype-Tissue-Expression (GTEx) project^52^.

The combination of analysis of -omics data and structural methods used in this study, expanding a framework that we recently applied to other proteins^46,53^, provide a detailed assessment of the different effects that ULK1 mutations can cause to perturb the protein structural stability or activity. Our results can guide the selection of ULK1 as a target in certain cancer types, suggest which readouts to study for experimental research in cancer cellular biology, and provide knowledge for pharmacological or clinical-oriented efforts.

## Results and discussion

### Alterations in gene expression in ULK1 and ULK2 in different cancer types

**Figure 1:**
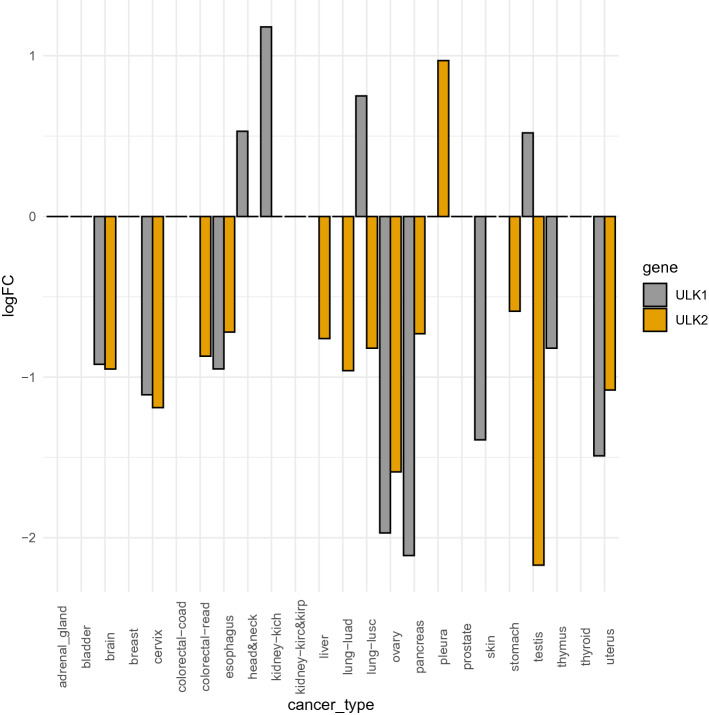
Changes in gene expression for ULK1 and ULK2 in the cancer datasets under investigation. In the plot, we report the Log Fold Change (logFC) comparing tumor versus normal samples, as estimated by *limma-voom* and including in the design matrix information on the Tissue Source Site (TSS). If the logFC > 0.05 the gene is considered up-regulated in the tumor with respect to the normal samples, whereas if the logFC is < − 0.05, the gene is down-regulated. When the logFC is set to 0 in the plot, it means that the gene is not differentially expressed according to Differential Expression Analysis (see Materials and methods for more details). The full list of logFC values is reported, together with the sample size and detailed information on the cancer datasets in Table S1.

In our study, we focus on the effects of mutations found in cancer samples in ULK1 kinase domain, since it is the only region with an available experimental structure. The majority of the rest of the protein has a high content of predicted disorder according to *FELLS*^54^. Nevertheless, due to the complexity of alterations occurring in tumors, it is also important to evaluate whether other alterations are occurring, such as at the level of expression. We analyzed the changes in gene expression of ULK1 using RNASeq data from TCGA^49,55^ and Recount2^51,56^, for a total of 30 different cancer types (Fig. 1, Table S1). Due to the high functional redundancy of ULK1 and ULK2^57^, we also monitored the changes of ULK2 in the same cancer types. We used differential expression analyses to estimate the changes in expression of all the genes in the dataset and then retrieved the estimate for ULK1 and ULK2 (Fig. 1). We observed different changes depending on the cancer types. Brain, gynecological and esophagus cancer types feature a downregulation of both genes, suggesting an impairment of their function in autophagy. In another group of tumors, compensatory effects might be in play with one of the two ULK genes downregulated and the other upregulated, as exemplified by Lung Squamous Cell Carcinoma, LUSC (Fig. 1). In other cases, only one of the two kinases is up- or downregulated, suggesting that the unaltered levels of the remaining kinase can have a partial compensatory effect. We did not observe marked changes in expression of either ULK1 or ULK2 in eleven of the cancer types used in our study, such as breast, bladder, and kidney.

### Functional elements of ULK1 kinase domain of interest for the study

**Figure 2:**
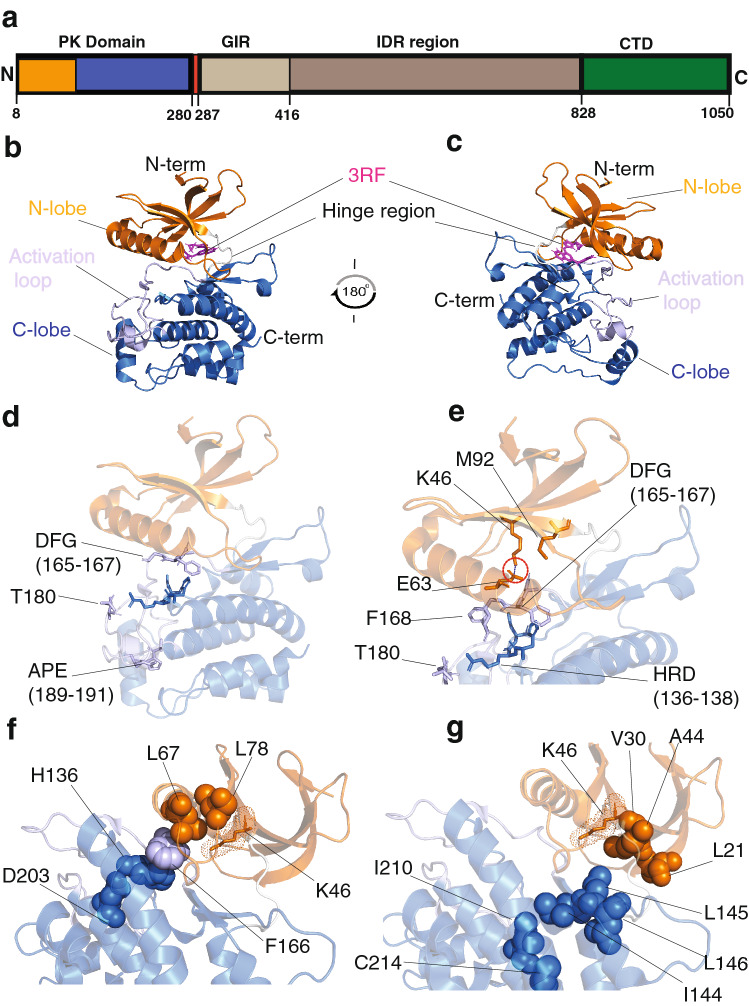
Structural and functional element of the ULK1 kinase domain, described by similarity with the c-SRC kinase. (**a**) A schematic view of the ULK1 domains. The numbering refers to the UNIPROT ID O75385. The domains are: (i) the protein kinase domain (8–280), the GABARAP interacting regions (GIR, 287–416), a predicted disordered region (416–828) and the C-terminal domain (828–1,050). We then highlighted on the structure of the ULK1 kinase domain (PDB entry 4WNO) important elements of kinase function. (**b,c**) The N- (residues 8–92) and C- (residues 99–280) lobes are shown in orange and blue, along with the hinge region (93-EYCNGG-98) of ULK1 in white. The region of the activation loop (163–200) is highlighted in light blue, along with a stick representation for the side chains of the phosphorylation site T180, the DFG and APE motifs. The inhibitor 3RF is highlighted in blue and pink. Panel C provides a 180 degrees rotation to show the opposite sites. (**d**) T180 and DFG (165–167) and APE (189–191) motifs. (**e**) The catalytic lysine (K46) and its salt bridge with E63 in the N-lobe are shown as orange sticks, along with the gatekeeper methionine (M92). The salt bridge is also maintained in all the structures sampled by the MD simulations collected in this study (see GitHub repository). F168 adjacent to the DFG motif, which is likely to be important for substrate specificity, along with the HDR motif are also highlighted as sticks. The residues of the aligned regulatory spine (**f**) and of the catalytic spine (**g**) are shown as spheres. The catalytic lysine with dots and sticks as a reference of the active site.

The ULK1 kinase domain (residues 8–280, Fig. 2a) consists of catalytic and regulatory regions and belongs to the CAMK (Ca^2+^/calmodulin-dependent kinase) family based on a structurally validated alignment of the kinome^58^. It can be divided into a small N-lobe (residues 8–92), a hinge region (93-EYCNGG-98), and a large C-lobe (99–280), as shown in Fig. 2b,c. Before illustrating our analysis, we aim to orient the reader on the important functional ULK1 elements that we will recall in the discussion of the results. The structural background illustrated below is needed to appreciate the effects that the mutations found in cancer samples could exert on this protein.

ULK1 kinase domain includes a long and positively charged activation loop (165–174 and 178–191) that may play a role for substrate recognition and activity regulation (Fig. 2b,c). ULK kinases share the long disordered activation loop, a rare feature in the rest of the kinome^31^. The kinase domain can be activated by phosphorylation on Thr180 on the activation loop^9,59^ (Fig. 2d). The activation loop of ULK1 also includes the invariant kinase DFG motif (Asp165-Phe166-Gly167) and extends up to the APE motif (Ala189-Pro190-Glu191). The activation loop generally forms a cleft for substrate binding when the kinase is in its active state. The bound substrates form specific interactions with a conserved HRD motif (His136-Arg137-Asp138 in ULK1) (Fig. 2e). The active state generally exhibits a salt bridge between a conserved lysine in the β3 strand (K46 in ULK1) and a glutamate residue (E63) in the C-helix (48–69, Fig. 2e). This salt bridge is conserved in the structures from the MD ensembles collected in this study (see below). A basic patch around the acetylation site K162 is also important for protein activation^60^. The basic patch can be involved in interactions of ULK1 with membranes or ATG13, along with for the binding to its own C-terminal domain^31^*.*

ULK1 is known to prefer serine residues in the target substrates^61^, a trait that we predict related to the presence of the F168 as DFG + 1 residue (Fig. 2e), in agreement with the findings by Chen et al.^62^ that large hydrophobic DFG + 1 residues promote Ser phosphorylation.

Kinases often present a gatekeeper residue in the active site^63^, which corresponds to Met92 in ULK1 (Fig. 2e). Mutations of gatekeeper residues in other kinases are associated with the development of chemotherapeutic resistance^64^. Mutations in the gatekeeper position of kinases also improve inhibitors potency, which can be increased by large-to-small mutations at this site^65^. In this context, methionine (as observed in ULK1) is among the larger and bulky gatekeeper residues found in kinases so far. The mutation of a threonine gatekeeper to methionine is associated with the development of drug resistance in other kinases^66^. A glycine-rich loop in the proximity of the DFG motif (G25 and G23 in ULK1) is also important for kinase function^67^.

The regulatory (RS0: D203, RS1: H136, RS2: F166, RS3: L67 and RS4: L78, Fig. 2f) and the catalytic spines (L21, V30, A44, L145, I144, L146, I210 and C214, Fig. 2g) observed for other kinases are conserved in ULK1. A comparison of active and inactive regulatory spines (R-spines, RS) of kinases showed that the RS3 residue in the C-helix of the dormant enzyme is displaced and the spine not properly aligned when compared to the active enzyme^68^. The R-spine consists of residues from both the N- and C-lobes. The histidine of the HRD motif and the phenylalanine of the DFG motif also contribute to the R-spine formation. V30 and A44 of the ULK1 catalytic spine should be the residues for the binding with the adenine group of ATP, whereas one of the leucine residues is likely to be important for the interaction with the adenine base. The catalytic and the regulatory spines control catalysis by dictating the positioning of the ATP and the substrate, respectively. Thus, their proper alignment is necessary for the assembly of an active kinase.

### ULK1 microsecond dynamics

**Figure 3:**
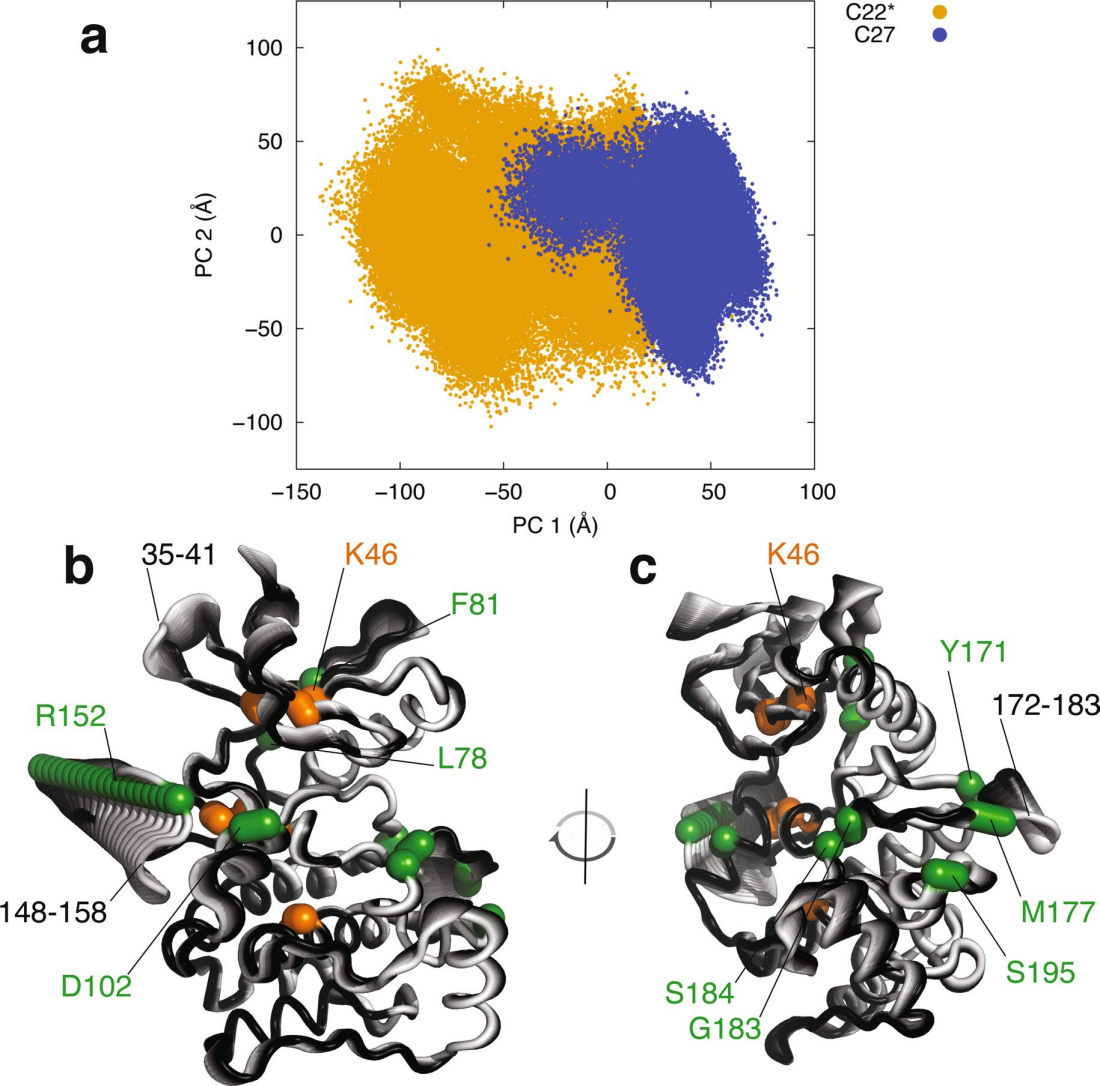
Principal component analysis of MD simulations of ULK1 kinase domain. (**a**) The overlap between the sampling described by the two simulations of ULK1 carried out with two different force fields is shown along the two first principal components as a visual reference. The two MD simulations show a certain degree of overlap and sampled similar regions of subspace described by the first two principal components (PC1 and 2), with some deviations as often encountered in comparisons of MD simulations of the same system with different force fields or even different replicates of the same system. (**b,c**) Conformational changes described by the first principal component, which interest three disordered regions of the protein with concerted motions. As a reference, the mutation sites in this area (discussed in the main text) and the residues important for ULK1 function are shown in green and orange, respectively.

Kinases are characterized by complex conformational changes and several dynamic elements^69,70^. Biomolecular simulations, which allow the description of protein dynamics over different timescales, proved their effectiveness in study structure–function-dynamics relationships in kinases^69^. Thus, we collected all-atom Molecular Dynamics (MD) simulations in explicit solvent to provide the first description of the ensemble of conformations of the ULK1 kinase domain in solution. An ensemble of conformations, as the one provided by MD, is also pivotal to the structural analyses required for the annotation of the ULK1 mutations found in cancer genomics studies, as we recently applied to other target proteins^46,53^. To rule out dynamic patterns that are depending from the physical model used in the simulations, we collected MD simulations of the ULK1 kinase domain with two different force fields (i.e., CHARMM22star and CHARMM27). Using a dimensionality reduction approach based on Principal Component Analysis, we compared the conformational sampling^71^ achieved with the two force fields (Fig. 3a). We find a good overlap in the subspace described by the two first principal components, which account alone for more than 40% of the atomic fluctuations, suggesting that the two simulations give a consistent view on the ULK1 dynamics. To quantify the overlap between the conformational space sampled by the two different force fields, we also calculated the root mean square inner product (RMSIP), which is a measure of the similarity of the structural space described by the first 20 principal components with a value of unity as an indicator of identical subspaces. We obtained a RMSIP value of 0.76, indicating a high similarity of sampled conformational subspaces, for the two simulations of the ULK1 kinase domain, confirming the results from inspection of the 2D projections.

Further, we analyzed the principal motions described by the first principal component (Fig. 3b,c). The analysis highlighted concerted motions between the loop 148–158 and a part of the activation loop (in the region 172–183), along with the loop adjacent to the catalytic lysine (in the region 35–41). The two disordered regions 148–158 and 172–183 feature motions of closure towards the rest of the ULK1 structure. The conformational change seems to be triggered by the electrostatic interactions between two arginine residues (R152 and R153) and a negatively charged residue (D102) on the facing helix. Simultaneously with this motion, a conformational change in the region 35–41 occurs where a loop moves apart from the L78 residue of the regulatory spine. The analyses of these dynamic patterns will be used in the annotations of the possible functional impact of ULK1 missense mutations found in cancer samples, discussed in the sections below.

### Missense mutations in cancer of ULK1 kinase domain

We retrieved the missense mutations in the coding region of ULK1 for each of the cancer studies deposited in TCGA (Table S2, Table 1). We identified the majority of the mutations in uterine, lung, colon, and stomach tumors (i.e., UCEC, LUSC, COAD and STAD). ULK1 is also predicted as a driver gene in some of these cancer types by *OncodriveCLUST*, a method based on positional clustering and exploiting the notion that variants in cancer-causing genes are enriched at few specific loci^72^. We should notice that these TCGA cancer types are characterized by a high mutational burden, as shown in a recent study^73^. Thus, it is not surprising that these tumor types have a higher number of missense mutations of ULK1.

**Table 1:** Summary of the missense mutations of ULK1 kinase domain analyzed in this study.

Mutation	TCGA study	Revel	CO-OCC. ULK2 mutations	DEA	SLiMs/PTMs
G12D	GBM	0.308	NONE	Down	
F14L	BLCA	0.205	NONE	No DE	
A28V	GBM	0.377	NONE	Down	
S56Y	UCEC*; COAD*	0.393	D73A;NONE	Down; no DE	
L78Q	STAD*	0.539	NONE	Down(ULK2)	
F81L	BLCA	0.215	R408S	No DE	
N86T	UCEC*	0.049	NONE	Down	
N96D	UCEC*	0.376	NONE	Down	
A101T	UCEC*	0.373	T102A	Down	
D102N	LGG	0.364	NONE	Down	
D113E	HNSC	0.318	NONE	Up(ULK1)	
A125T	COAD*	0.741	NONE	No DE	
I135V	BLCA	0.14	NONE	No DE	
R137H	STAD*	0.796	NONE	Down(ULK2)	
R137C	COAD*	0.843	NONE	No DE	Introduce *S*-nitrosylation
D138N	LUSC;PAAD	0.769	NONE;NONE	Down;down	
R152L	LUAD*	0.226	NONE	Down(ULK2)	
A154S	LIHC	0.06	NONE	Down(ULK2)	
G167A	LUSC	0.91	NONE	Down	
A169T	KIRP	0.751	NONE	No DE	
Y171H	SKCM	0.121	R951K	Down(ULK1)	
M177V	STAD*	0.632	NONE	Down(ULK2)	
G183V	LUSC	0.766	NONE	Down	
S184F	HNSC	0.797	NONE	Up(ULK1)	
S195P	UCEC*	0.531	NONE	Down	Predicted phosphorylation by DNAPK or ATM kinases
V211I	UCEC*	0.04	NONE	Down	
L215P	BRCA	0.716	NONE	No DE	
E246D	COAD*	0.187	R254I	No DE	
P250S	COAD*	0.082	NONE	No DE	Introduce phosphorylation by PKC kinase
R252L	LUSC	0.31	NONE	Down	
R252W	STAD*	0.31	NONE	Down(ULK2)	
R261C	STAD*	0.391	V868M	Down(ULK2)	
R261H	PAAD	0.345	NONE	Down	
D268H	OV	0.255	NONE		
F273V	UCEC*	0.37	R303C		
D279N	UCEC*	0.152	NONE		IAP-binding motif (279–283, ELM)

We report the mutation identified in TCGA data and located in the kinase domain of ULK1. The cancer studies in which ULK1 is predicted as driver genes are marked with a *. The full name of the TCGA cancer studies are reported in Table S1. We show also the results of the prediction of damaging or neutral mutations with *Revel.* The cutoff for damaging mutations, according to the *Revel* score, is set to 0.4. We also indicate the overlap with short interaction linear motifs (SLiMs) and post-translational modifications (PTMs). The original results of each predictor are reported in Table S2. In this table we used simplified and common labels across the predictors, i.e. damaging or neutral to allow a more straightforward comparison. We also reported as a reference the results of DEA (Fig. 1 and Table S1) for ULK1 and ULK2. ‘Down’, ‘up’ and ‘no DE’ indicate that the genes are down-, up-regulated, or not differentially expressed.

**Figure 4:**
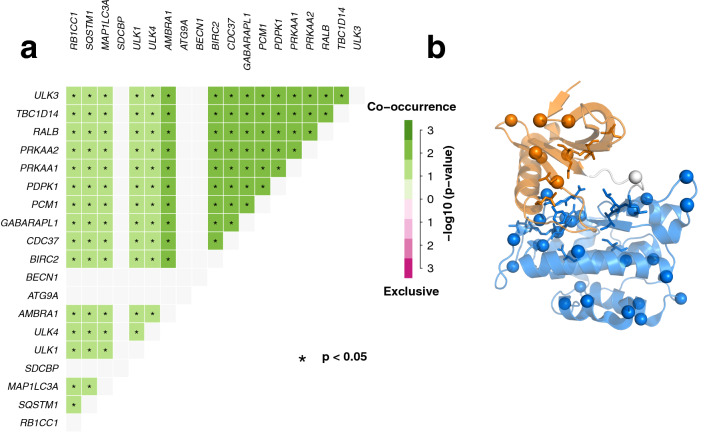
Missense mutations in the ULK1 kinase domain in TCGA datasets and co-occurrence of mutations in ULK1 interactome. (**a**) We used, as a description of the ULK1 interactome, the interactions annotated in *IID* and estimate the co-occurrence of mutations in the TCGA datasets under investigation. The results for the TCGA pancreatic cancer dataset are shown as an example. Empty lines indicates that no occurrence was found for the corresponding interactor. The other data are available in the GitHub repository associated to this publication. (**b**) The mutation sites are highlighted on the 3D structure, along with the sub-domain composition (N-, C-lobe and hinge in orange, marine and light grey, respectively). The residues of the DFG and HDR motifs are shown as sticks, whereas the catalytic lysine with sticks and dots.

ULK1 is a multi-domain protein with scaffolding functions and, as such, can interact with a multitude of different other proteins. For a proper assessment of ULK1 missense mutations, it is important to gain knowledge on the alterations, in the same tumor samples, of the biological partners of interaction. To this scope, we curated the ULK1 interactome, mining the *IID* protein–protein interaction database^74^. We then estimated the co-occurrence of mutations among each of the 30 identified interactors and ULK1 mutations for each cancer type (Table S2). We found co-occurring mutations between ULK1 and its interactors in eleven cancer types in which ULK1 has been found mutated (see GitHub repository for more details on each of them). Among these, stomach, skin, brain, colorectal, uterine and pancreas tumor samples (Fig. 4a, as an example) are characterized by co-occurring mutations in a large number of members of the ULK1 interactome, especially the ones important for the upstream regulation of autophagy For example, we found AMBRA1, components of the mTOR and ULK1 complex, RB1CC1/FIP200, TBC1, AMPK subunits, members of the ATG8 family, ATG16L1, BECN-1, PDPK,I RGM, RAB1A, P62, MINK1, SDCBP, and ATG13). This suggests a pronounced effect of alterations related to ULK1 function and activity, and, more in general, upstream steps of autophagy, in these cancer types.

We also verified if mutations of ULK2 kinase domain were co-occurring with mutations in ULK1, and we observed this pattern only in few isolated cases (Table 1). The mutations occurring in ULK2 kinase domain (D73A, T102A, R254I) are predicted with no effect on protein stability with the methods illustrated below, resulting in minor changes of free energy associated with stability (i.e., 0.2–1.6 kcal/mol). In most cases, we noticed that most of the mutations occurred in cancer types where ULK1 and ULK2 gene expression is down-regulated.

### Mutations in the catalytic domain of ULK1

As mentioned above, the kinase domain of ULK1 is the only part of the protein for which a structure is available and which allows us to use other methods to assess the impact of mutations, beyond the observation of co-occurrence.

In total, we collected 36 different missense mutations of ULK1 kinase domain distributed over the whole structure (Fig. 4b) of which D138N of the HRD motif occurred in both pancreatic and lung cancer samples and, the R137, R252, and R261 sites were mutated to different residues in different cancer types.

We then turned our attention to a workflow similar to the one that we recently applied to another protein^53^ for a more comprehensive assessment and understanding of the effects induced by ULK1 mutations. We evaluated different properties to discriminate between effects associated with the structural stability of the protein or with its function. Moreover, these analyses help to link the effects of the mutations with specific factors for ULK1 activity or regulation, which is an important piece of knowledge to guide the experimental characterization of the molecular mechanisms and phenotypes associated with each mutation. The analyses used for the assessment are described one by one in the following sections, for the sake of clarity.

### Interplay of the ULK1 mutations with post-translational modifications and functional motifs

As the first factor for our assessment, we evaluated each mutation site in the context of interplay with post-translational modifications (PTMs) and overlap with functional short linear motifs (SLiMs), along with the potential of harboring new PTM sites upon mutation (Table 1). These are properties that can ultimately influence the regulation of the protein or its spectrum of interactions.

Moreover, we evaluated if any additional mutations was found in the LC3 interaction region (LIR) of ULK1^75–77^, which is placed in a distal region with respect to the kinase domain. This analysis was motivated by the observation that mutations in members of the ATG8 family are co-occurring with mutations of ULK1 in some of the TCGA cancer studies (Fig. 4b). LIRs are SLiMs for interaction between the LC3/GABARAP (ATG8) family members and other autophagy proteins and key mediators of autophagosome formation^78^. We recently found mutations of LC3B co-occurring with mutations in its LIR-containing interactors in cancer genomic data^53^, and we thus aimed to evaluate if the same happens for ULK1. We did not find any mutations in the surrounding of the ULK1 LIR region in the TCGA samples under investigation, thus suggesting that the recognition between ULK1 and the ATG8 family is not a major driver of its alterations in these cancer samples.

The only mutation in the proximity of a SLiM is the C-terminal D279N, which includes an IAP (Inhibitor of Apoptosis Protein) binding motif (IBM). This motif has not been characterized in ULK1 yet, at the best of our knowledge. Interestingly, one of the ULK1 interactors, BIRC2 (Table S2) is a cellular inhibitor of apoptosis and promote autophagy, interacting with ULK1 during mitophagy^79^. We speculate that this interaction could be mediated by the IAP motif of ULK1 and that the mutation of D279N could impair it. The mutations have been found in uterine cancer, where mutations of ULK1and BIRC2 are co-occurring (see GitHub repository).

We did not identify any mutations directly altering an experimentally validated PTM site. Nevertheless, we find one solvent-exposed mutation site (S195) which is predicted as a phosphorylation site for DNAPK or ATM kinases. A substitution to proline could have the effect to abolish this modification. We then analyzed the mutations to serine, threonine or tyrosine for their capability to introduce new phosphorylatable residues, along with mutations to cysteine for their possibility to introduce a redox-sensitive post-translational modification, i.e., *S*-nitrosylation^80^ (Table 1, Table S2). P250S ULK1 could result in a phosphorylatable serine by PKC kinase and the R137C mutation in the HRD motif could introduce a possible *S*-nitrosylation site.

### Assessment of the impact on ULK1 protein stability of the missense mutations

One of the main effects that a mutation can have on a protein is to alter its structural stability, causing local misfolding and a higher propensity for the mutated variant to be targeted by pathways for protein clearance, such as proteasomal degradation^42,81,82^. In this scenario, the function of the protein will also be affected, but mostly as the result of compromised protein levels and not necessarily an alteration of its capability to interact with the biological partners or be an active enzyme.

We used a high-throughput saturation mutagenesis approach^83^ based on an empirical energy function implemented in *FoldX* to predict the effect on protein stability induced by all the possible substitutions of each position of the structure of the kinase domain of ULK1. This approach has the advantage of providing both the estimate of the damaging effect of the disease-related mutation of interest and a pre-computed list of predicted changes in stability for any other mutations of the protein. The latter is useful to identify important hotspots for the structure of ULK1, along with pre-annotated effects of amino acid substitutions that can be consulted for newly discovered mutations in future genomics studies.

To overcome the inherent issues in local sampling and lack of backbone flexibility of *FoldX*, we used the MD-derived ensembles for the analysis. We estimated the changes in the free energy of folding upon mutation (Tables S3–S6), as recently applied to other cases study^46,53,84^. The predicted ΔΔGs, using the two different MD ensembles of the ULK1 kinase domain, are in good agreement (Table S3). Moreover, we notice that, for some mutations, the predicted damaging effect is a result of the usage of the static X-ray structure (Table S3), whereas the possibility to account for the flexibility of the protein structure in the prediction result in neutral effect. An example of this behavior is A28V (Table S3). Based on this observation, we used the ΔΔGs predictions from the calculation on the MD ensembles to classify the ULK1 mutations found in cancer patients (Fig. 4b). Two mutations (S184F and V211I) could stabilize the protein architecture, suggesting a better packing of the protein. S184 is located in the proximity of aromatic residues, including the one of the DFG motif, which is important for catalysis so it cannot be excluded that a mutation to S184F could alter the functional dynamic of the kinase at this site.

**Figure 5:**
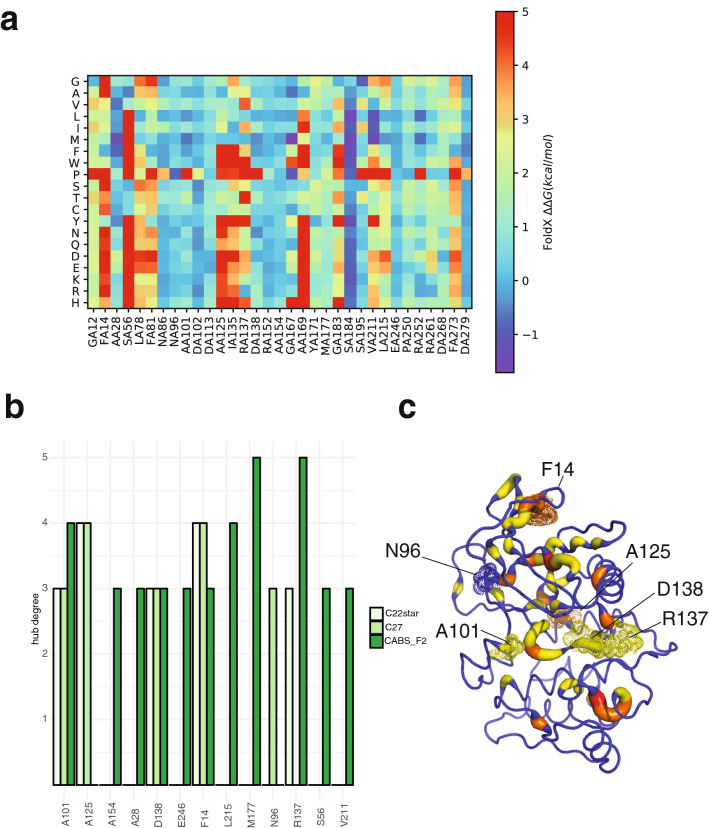
ULK1 mutations in TCGA cancer samples altering protein stability. We used two different estimates of the impact on protein stability, based on the calculations of changes in free energy of unfolding with an empirical scoring function (**a**) and the notion of hub residues in a Protein Structure Network (**b,c**). (**a**) A heatmap with the estimated changes in free energy upon a deep mutational scanning of all the mutation sites is reported. As an example, we showed the results for the MD-ensemble with CHARMM22star force field. The results of the other mutational scans are reported as Tables S4-S6. More than 50% of the ULK1 mutations are predicted to destabilize the protein structure and located in sites that are general hotspots for maintenance of the native fold. (**b**) Hub prediction are in fair agreement using different methods to generate the ensemble of conformations, i.e. MD simulations with two different force fields (CHARMM22star and CHARMM27), along with a coarse-grained sampling method with a posteriori reconstruction of a full atom model (CABS-Flex), which overestimate some of the hubs in the C-terminal domain of the protein. (**c**) Hub-behavior of the mutation site in the MD ensemble with CHARMM22star force field, as an example.

We also notice that some mutation sites are more general hotspots for ULK1 structural stability (Fig. 5a). In these cases, the sites are sensitive to substitutions to most of the other residues (i.e., F14, S56, L78, F81, A125, R137, A169, G183, and F273). I135 is also a stability hotspot, but the I135V mutation, which was found in the cancer samples, is one of the few tolerated substitutions, suggesting a neutral effect.

Next, the availability of MD ensembles for ULK1 kinase domain prompted us to apply a Protein Structure Network (PSN) approach based on the persistence of side-chain contacts in the conformational ensemble^85,86^ to estimate hub residues, which are often corresponding to important residues for protein stability (Fig. 5b,c). We verified which of the mutation sites are found in correspondence or proximity of a hub, as an additional parameter to evaluate the impact of the substitution on protein stability. Moreover, to account for the fact that the mutation in a hub can still retain its hub capability, we collected the same analyses on conformational ensembles derived for each of the mutant variants (Table S3). We classified as damaging mutations according to this parameter only the ones for which the substitution abolishes the hub behavior. We find only one case, i.e., F14L, where the hub behavior is conserved upon mutation. Most of the mutation sites in PSN hubs also correspond to mutational hotspots associated with high ΔΔGs for protein stability, except for D138N.

Another strategy to impair structural stability could be related to the loss of electrostatic interactions in the form of salt bridges or hydrogen bonds. We calculated the persistence of the salt bridges and their network in the MD ensembles and annotate which of the mutation site where likely to abolish these interactions (Table 2). Most of the mutations of residues involved in salt bridges are likely to have marginal effects since they conserve the negatively charged nature of the wild-type residue or they are replaced by asparagine, which could still account for electrostatic interactions with the guanidinium group of arginine. The only mutations which could impair salt-bridge formation are R152L and D268H. R152L is involved in salt bridges in the CHARMM27 MD ensemble, whereas it shows a loose tendency to form electrostatic interactions in CHARMM22* MD ensembles. MD force fields are known to have limitations in overestimating salt bridge contributions^87,88^, and according to a recent benchmarking^88^, we selected the CHARMM22star results for the annotation of the mutations with respect to salt-bridge formation. 58%of the mutations of the ULK1 kinase domain found in the different cancer types are predicted damaging for protein stability using at least one of the two criteria above.

**Table 2:** Salt-bridges involving ULK1 mutation sites characterized by charged residues, along with their persistence in the MD ensemble.

Mutation	CHARMM22star (%)	CHARMM27 (%)
D102N		R152-D102-R153(67.5, 81.0)
D113E	D113-R116 (44.3)	D113-R116(55.8)
D138N	D138-K140 (99.8)	D138-K140(99.8)
R152L		R152-D102(67.5)
E246D		E246-R245(22.7)
D268H	D268-K201(98.8)	D268-K201(93.0)
D279N	D279-R116(63.2)	D279-R116(59.4)

R137, R252 and R261 are not reported since we did not find any stable salt bridges involving these residues in the simulations.

### Assessment of the impact of mutations on ULK1 function

The availability of structure and dynamics of ULK1 allowed us to predict effects induced by the mutations on its function. We evaluated the occurrence of the mutations in proximity of the disordered regions interested by the functional motions underpinned by Principal Component Analysis (Figs. 3b,c, 5b,c). R152L and D102N are likely to impair or weaken, the functional motions observed for the region 148–158, which are triggered by their electrostatic interactions. We cannot rule out that the presence of R153 in the R152L mutant variants could partially compensate for the mutation. On the other side, different mutation sites are in the area of the lateral motion of the region 172–183 of the activation loop, such as M177 in the loop itself, S195 in the region where the loop bends, and Y171, G183/S184 which act as hinges for the motion of these regions. It could be expected that these mutations impair ULK1 functional dynamics. In addition, the regulatory spine residue L78 and F81 in the proximity of the other disordered loop (35–41), which is involved in the concerted conformational changes, are also corresponding to mutation sites.

**Figure 6:**
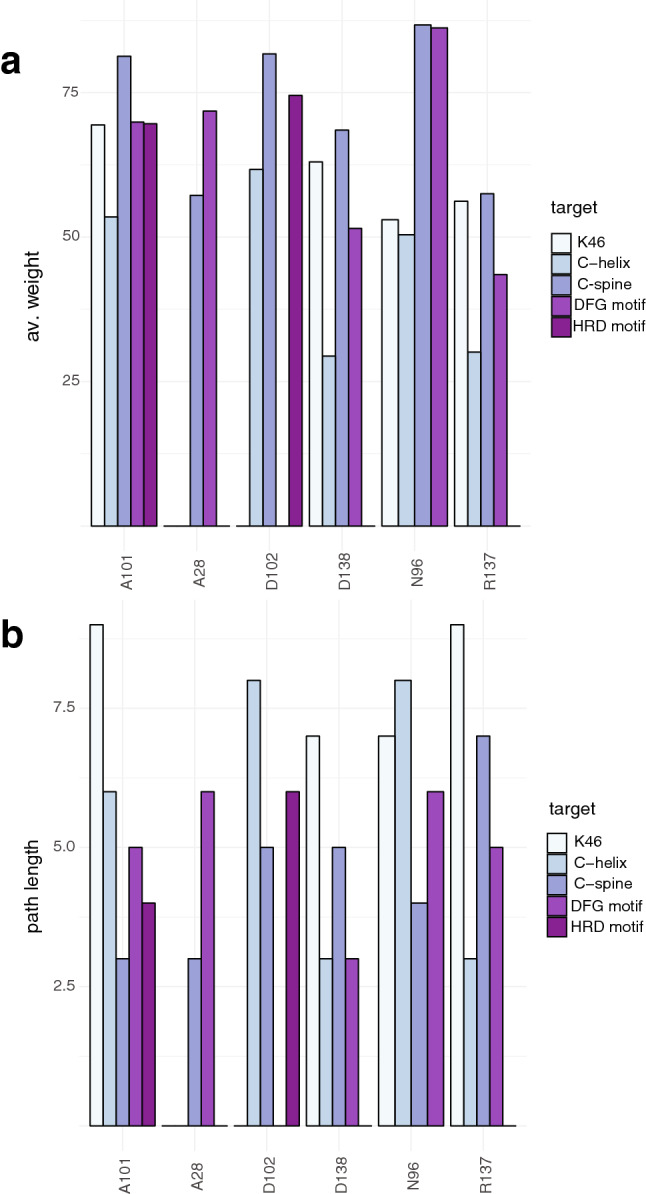
Shortest paths of communication between the mutation sites and key functional or regulatory regions of ULK1 kinase domain. The whole set of results is available in Table S7. We reported the data for CHARMM22star MD simulations for sake of clarity, which were very similar to the ones for the simulation carried out with CHARMM27. If a mutation site featured more than one path of communication for the same class of target residues, only the path with lower path length is taken into account in this figure. (**a**,**b**) report the average weight and the path length, respectively We used as target residues: (i) residues important for activity (K46, E63, M62, T180 and K162); (ii) residues of the DFG, HRD, and APE motifs; (iii) central residues of the C-helix (55–65); (iv) the residues of the catalytic and (v) regulatory spines. No paths were found from the mutation sites to the regulatory spine or the APE motif in both the MD simulations.

PSN approaches can be used to infer functionally-damaging sites if the paths of communication between the mutation sites and other important functional sites for the kinase activity are considered. This analysis can shed light on effects that are likely to be transmitted long-range, often at the base of allostery^45,47,89^. We estimated all the shortest paths of communication between each mutation site and five important classes of residues for kinase function. In particular, we selected three groups of target residues: (i) residues important for activity (K46, E63, M62, T180 and K162); (ii) residues of the DFG, HRD, and APE motifs; (iii) central residues of the C-helix (55–65); (iv) the residues of the catalytic and (v) regulatory spines (Fig. 6a,b). We selected only those paths conserved both in the CHARMM22star and CHARMM27 simulations (Table S7). We did not find any communication roads to the regulatory spine or the APE motif. On the contrary, a subset of mutation sites (A28, A101, D102, D138, N96, and R137) was communicating with at least two of the target areas of interest, often using multiple paths. This result suggests that substitutions at these sites could be detrimental for functional long-range communication or it could increase it (if oncogenic). This could be especially the case in which mutations alter the steric hindrance or the physicochemical properties of the wild-type residue, as in the case of A28V, A101T, R137C and, R137H. We used the statistical mechanical model implemented in AlloSigMA^90^ to have a more direct proof that these mutations could exert an allosteric effect (see GitHub repository for the outputs).

### General assessment and classification of ULK1 missense mutations in TCGA

**Figure 7:**
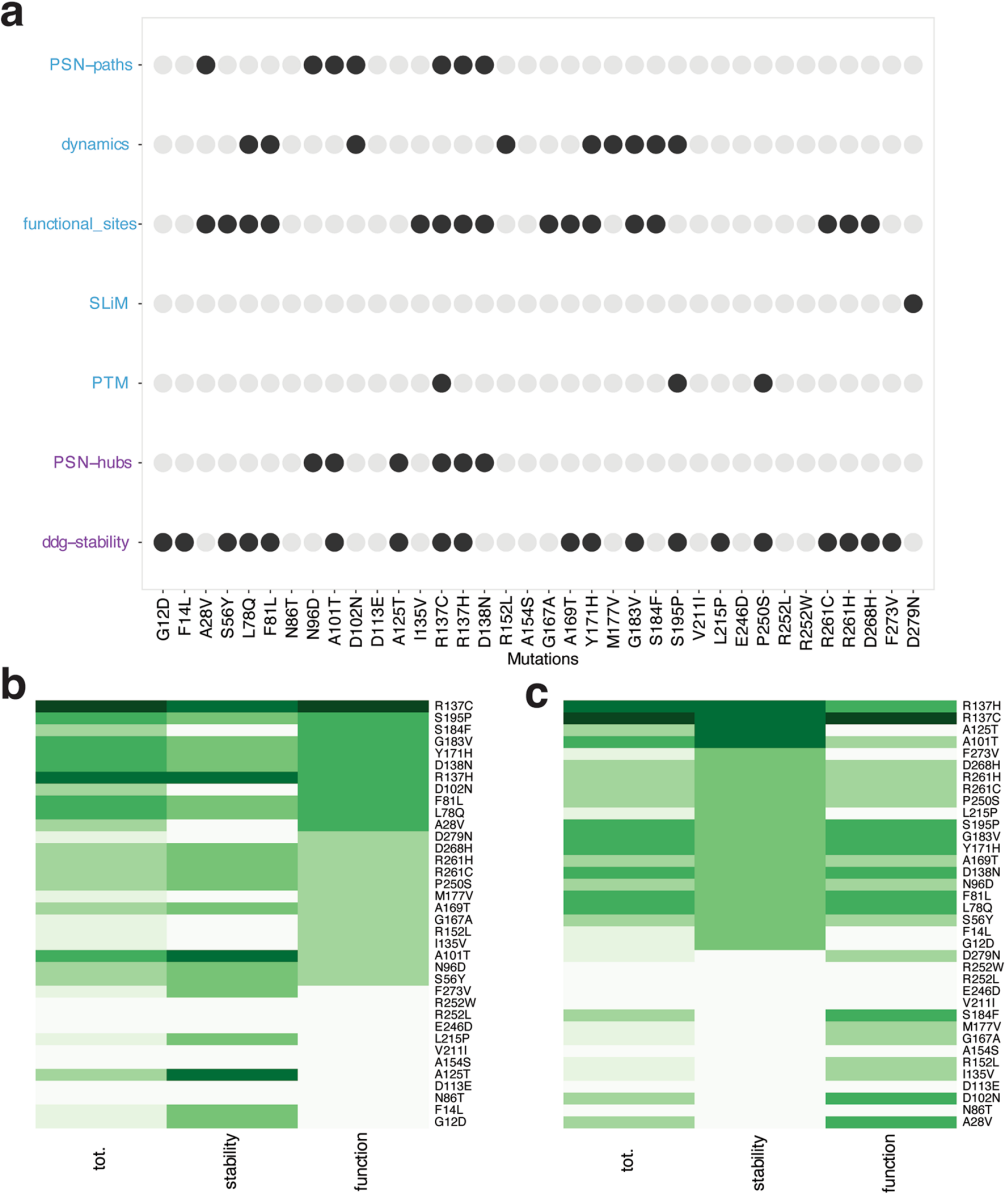
Classification of ULK1 missense mutations found in cancer genomics studies. (**a**) The different analyses used in this study have been aggregated to associate the potential of damaging or neutral effect of each mutation. We used descriptors that account for either protein stability (purple) or function (blue). Mutations altering one of these properties are indicated as black dots. The diagram allows to link each mutation with a specific effect which could also guide the selection of the more appropriate set up for experimental validation. (**b,c**) The heatmaps with the results of ranking on a collective score of damaging potential for the mutations in term of function (**b**) or stability (**c**). Darker the color more damaging the mutation is predicted to be. The values range from 0 to 5 and from 0 to 2 for function and stability, respectively.

We integrated all the results collected by each of the analyses above to provide an overall view on the several properties and layers of alterations that a mutation in a protein can cause, and which are ultimately connected to the alterations in its function at the cellular level. In particular, with our framework we can assess: (i) effects on protein structural stability, which will impact on the protein levels and turnover in the cell; (ii) interplay with post-translational modifications and emergence of new layers of regulation; (iii) alterations of binding regions for biological partners; and (iv) long-range functional effects. We classified the mutations according to each of these properties as damaging or neutral (Fig. 7a) and then ranked them. The ranking allowed us to identify mutations that are likely to be damaging, along with identifying if the effect is triggered more by destabilization of the protein product or a stable protein variant with impaired functionality (Fig. 7a,b).

As stated above, we observed that more than 50% of the mutations of ULK1 kinase domain found in the cancer samples are predicted to alter protein stability. This is often accompanied by a possible impact also on the native functional properties of the same variant. A minority of mutations are only damaging for stability (A125T, F273V, L215P, F14L and G12D) and do not alter the functional state of the protein. The detrimental effect on the protein stability observed by these mutations could alter the cellular level and turnover of the protein. This effect could be dominant with respect to the effects that the same mutations exert on protein activity or interactions.

ULK1 stability could also be modulated by the interaction with ATG13 and FIP200, which bind to the cytoplasmic domain of the ULK1^37^, or by chaperonin-like proteins, such as p32^91^. Thus, these interactions might compensate for the loss of stability induced by some of the mutant variants. Interestingly, in several cases, the cancer types where destabilizing mutations of ULK1 occurred also feature co-occurrence of mutations in ATG13 or FIP200 (Table S3), suggesting an overall alteration of ULK1 stability. Three mutations (S184F, D102N, and A28V) are predicted with a possible impact only on kinase activity, either altering the functional dynamics of the protein or the capability to exert long-range effects from distal site to the functional and catalytic regions.

We searched in the literature if the mutation sites under investigation have been subjects of experimental studies and if the results of these experiments corroborate our prediction. Most of the mutations that we found are related to the mouse variant of ULK1, which shares 97.5% of sequence identity with the human variant in the catalytic domain. We found a study reporting a mutation of S184 to alanine^92^. S184 is mutated to phenylalanine in head and neck TCGA samples and, we predict a marginally stabilizing effect upon mutation. Moreover, we classified S184 as a functional damaging mutation likely to impair ULK1 functional dynamics, due to its hinge behavior for the motions of the activation loop. S184A and S184D have been reported to inactivate the ULK1 kinase^92^, supporting the predicted functional role more than an effect on structural stability. We collected other mutations at other sites for which the functional impact has been studied experimentally and they are summarized in Table S3. Of interest, S174A mutation results in a hyperactive enzyme^92^ and it is located in the region of the activation loop undergoing conformational changes. Moreover, we notice that the other experimental mutations with a reported effect on ULK1 activity are predicted to have marginal effects on protein stability, except for K46N, M92A, and Y89A. These mutations result either in inactivation of the kinase^93^ or impairment of phosphorylation of the ATG13 substrate^25^. Our calculations suggest that the detrimental effect could be due to changes in protein stability, an aspect which could deserve further investigation to verify the cellular levels and half-life of these variants to conclude which one is the predominant effect.

## Materials and methods

All the inputs, scripts and main outputs of this study are available in the GitHub repository https://github.com/ELELAB/ULK1_mutations. The trajectories and input files for the molecular dynamics simulations have been deposited in OSF: https://osf.io/8xuaj.

### Expression levels of ULK1 in TCGA datasets

We downloaded and pre-processed level 3 harmonized RNA-Seq data (HTSeq count) for all the available datasets from TCGA. We downloaded the data in June 2019 from the Genomic Data Common (GDC) Portal using the GDCdownload function of *TCGAbiolinks*^94^. An overview of the analysed datasets is reported in Table S1. We employed the *TCGAbiolinks* function *GDCprepare* to obtain a Summarized Experiment object^95^. We removed outlier samples with the *TCGAanalyze_Preprocessing* function of *TCGAbiolinks* using a Spearman correlation cutoff of 0.6. We normalized the datasets for GC-content^96^ and library size using the *TCGAanalyze_Normalization* function of *TCGAbiolinks*. Lastly, we filtered the normalized RNA-Seq data for low counts across samples using the function *TCGAanalyze_Filtering* with a 0.20 cutoff for quantile filtering. For the TCGA datasets where normal samples were missing, we used the unified dataset that integrates the Genotype-Tissue Expression (GTEx) datasets^52^ of healthy samples and the TCGA data, as provided by the *Recount2* protocol^51^. We also employed this dataset as an additional source of information for the TCGA datasets with less than five normal samples (see Table S1). We used the *TCGAquery_Recount2* function of TCGAbiolinks^97^ to query the GTEx and TCGA unified datasets. We carried out GC-content normalization and quantile filtering on the unified datasets, as described above.

Differential expression analyses have been carried out using *limma-voom*^98^ as implemented within the *TCGAanalyze_DEA function* of *TCGAbiolinks*^97^, along with *edgeR* to confirm the results, as we recently applied to another case study 100. We included in the design matrix conditions (tumor vs normal) and the TSS (Tissue Source Site; the center where the samples are collected) or the Plates (where available) as source of batch-effects to assess the robustness of the estimate of changes in expression with respect to different correction factors. In all our DEA analyses, we defined as a cutoff to retain significant DE genes a log fold change (logFC) ≥ 0.5 or ≤ − 0.5, whereas a cutoff of 0.05 was used for the False Discovery Rate (FDR). We then retrieved the estimate logFC for ULK1 (ENSEMBL ID ENSG00000177169) and ULK2 (ENSG00000083290) in the different comparisons (see Table S1).

### Curation and analyses of missense mutations of ULK1 from TCGA

We retrieved mutations for ULK1 from each TCGA cancer study using the *MuTect2* pipeline^100^ as implemented in the *TCGAbiolinks* function *GDCquery_Maf*. We retained missense mutations in the kinase domain of ULK1 for the structural analysis. For each mutation, we also collected the following additional information: (i) *REVEL* score^101^; (ii) interplay with post-translational modifications and functional short linear motifs using as a source of information *PhosphoSite*^102^ and *ELM*^103^, respectively; (iii) identification of the same mutation in *COSMIC*^104^. We also evaluated if the mutations under investigation were not found in *ExAC*^105^. as natural polymorphisms with high frequency in the healthy population and as a such, not interesting in a cancer context. We also used *iSNO-AAPair*^106^*, SNOSite*^107^ and *NetPhos*^108^ to predict *S*-nitrosylation or phosphorylation sites upon mutation to cysteine or phosphorylatable (serine, threonine and tyrosine) residues, respectively. We used the *NetPhos* predictor only for those mutations that were in solvent exposed sites upon analyzes of solvent accessibility of their sidechain with *NACCESS* (https://wolf.bms.umist.ac.uk/naccess/). Moreover, we verified that each mutation under investigation was the only one targeting the ULK1 gene in the sample where it was identified.

### Interactome of ULK1 and co-occurrence of mutations

We retrieved the experimentally known ULK1 interactors through the *Integrated Interaction Database* (IID) version 2018-05^74^. We then estimated the co-occurrence of mutations between ULK1 and each of these interactors, along with other ULKs kinases (i.e. ULK2, ULK3 and ULK4) with the *somaticInteractions* function of *maftools* R/Bioconductor package^109^, which performs a pairwise Fisher’s Exact test to detect significant pairs of genes.

### Prediction of driver genes

We used the *oncodrive* function of *maftools*^109^ to evaluate if ULK1 was predicted as driver gene in any of the cancer type under investigation. The function is based on the algorithm *oncodriveCLUST*^72^.

### Free energy calculations

We employed the *FoldX* energy function^110,111^ to perform in silico saturation mutagenesis. Calculations with this empirical energy function resulted in an average ΔΔG (differences in ΔG between mutant and wild-type variant) for each mutation over five independent runs performed using: (i) the X-ray structure of ULK1 (PDB entry 5CI7^32^), (ii) an ensemble of 20 representative conformations from the MD simulations with CHARMM22star or (iii) with CHARMM27. The protocol is detailed in our previous publication^46^. We also performed a literature-based curation of mutations for which the effects have been studied experimentally and use them as a control of the quality of our predictions.

### Molecular dynamics simulations

We carried out 1-μs molecular dynamics (MD) simulations for the human ULK1 kinase domains in explicit solvent using GROMACS software version 4.6^112^. We used as starting structure the PDB entry 5CI7 after in silico retro-mutation of the phospho-Thr 180 to Thr, to provide a model of the unphosphorylated variant of the domain. We used two protein different force fields CHARMM22*^113^, CHARMM27^114^ in combination with the TIP3P water model^115^ to evaluate the robustness of our results with respect to different physical models.

We used a dodecahedral box applying periodic boundary conditions and a concentration of NaCl of 150 mM, neutralizing the charges of the system. The simulated system (protein + water) accounted for 83,077 atoms. The system was prepared by different steps of minimization, solvent equilibration, thermalization and pressurization. We carried out productive MD simulations in the canonical ensemble at 300 K using velocity rescaling with a stochastic term^116^. We applied the LINCS algorithm^117^ to constrain the heavy atom bonds to use a time-step of 2 fs. We calculated long-range electrostatic interactions using the Particle-mesh Ewald (PME) summation scheme 119, whereas we truncated Van der Waals and short-range Coulomb interactions at 10 Å.

We verified the absence of artificial contacts between the periodic images of the protein in the simulations, which were always at a distance higher than 30 Å. We evaluated the quality of the conformational ensemble on 100 representative conformations equally spaced in time for each of the simulations using the machine-learning based approach implemented in *ResProx*^119^ to predict the atomic resolution from structural ensembles of proteins. We obtained a predicted resolution of 1.53 ± 0.30 and 1.66 ± 0.12 Å for CHARMM22star and CHARMM27 simulations of ULK1 kinase domain, respectively. These values are very close to the resolution of the corresponding X-ray structure (1.74 Å), suggesting an overall quality of the MD-based ensemble.

We used Principal Component Analysis (PCA) of the covariance matrix of Cα atomic fluctuations to extract the principal motions from the MD simulations^120^. We performed the PCA on a concatenated trajectory of the two MD simulations of ULK1 to compare them in the same essential subspace.

### CABS_flex ensembles of selected ULK1 mutant variants

For a selection of mutant variants of ULK1 (F14L, N96D, A101T, A125T, R137C, R137H and D138N) that have hub-behavior, we also collected conformational ensembles using the coarse-grained approach implemented in *CABS_flex* 2.0^121^.

We used as starting structures for *CABS_flex* calculations the models generated by *FoldX* during the mutational scan for each of these mutations. In particular, we selected the most representative models in terms of rotameric state of the mutated residue for each mutation. We then collected an ensemble of ten different conformations for each mutant variant to be used for the contact-based PSN analyses of hub residues described below, upon reconstruction of the corresponding full-atom models.

### Protein structure networks

We employed a contact-based Protein Structure Network (PSN) to the MD ensemble as implemented in *Pyinteraph*^85^. We defined as hubs those residues of the network with at least three edges^43^. We used the node inter-connectivity to calculate the connected components, which are clusters of connected residues in the graph. We selected 5 Å as the optimal cutoff for the contact-based PSN using the *PyInKnife* pipeline^86^. The distance was estimated between the center of mass of the residue side chains. We removed spurious interactions during the simulations applying a persistence cutoff of 20% (i.e., each contact was included as an edge of the PSN only if occurring in 20% of the MD frames), as indicated in the original implementation of the method^85^. We applied a variant of the depth-first search algorithm to identify the shortest path of communication. We defined the shortest path as the path in which the two residues were non-covalently connected by the smallest number of intermediate nodes.

We also calculated the persistence of salt bridges and hydrogen bonds with *PyInteraph* and the corresponding networks. For salt-bridges, all the distances between atom pairs belonging to charged moieties of two oppositely charged residues were calculated. The charged moieties were considered as interacting if at least one pair of atoms was found at a distance shorter than 4.5 Å. In the case of aspartate and glutamate residues, the atoms forming the carboxylic group were considered. The NH3- and the guanidinium groups were employed for lysine and arginine, respectively. We also verified the consistency of the results with a cutoff of 5 Å. We applied a persistence cutoff to filter interactions of 20% also for these networks.

## Conclusions

The assessment of the different effects that a mutation can exert on a protein explored in this study and the subsequent classification of the mutations can provide a useful complement to cancer genomics studies. For example, it allows to identify mutations that are likely to be ‘driver’ or ‘passenger’, along with to predict if the effect is triggered more by a destabilization of the protein product or a protein variant with impaired functionality. Moreover, our combined approach for mutation assessment could also benefit for the prioritization and selection of mutant variants for cellular experimental validation. Indeed, it can suggest how to select the proper readout for experimental validation. As an example, in a case where the mutation is predicted to be damaging for stability, experiments to estimate its cellular levels and half-life could be used, along with readouts to evaluate if the changes are due to proteasomal degradation or other clearance mechanisms. On the other side, if a mutation is predicted to result in a variant which is as stable as the wild-type, but the effect is more related to its function, experiments to evaluate its interactions in the cell with the biological partners, its regulation by PTMs or cellular assays to evaluate the effects on the pathways where interactors of the target protein are involved would be the most suitable choice. Moreover, we showed how the structural analyses used here benefit of the integration of bioinformatic tools to assess the changes in expression level of the target gene along with changes in other genes that can have compensatory effects, as we exemplified for ULK2. In addition, the extension of the analyses to the protein target interactome in terms of understanding co-occurrence of alterations and synergic effects that can arise from them allow a comprehensive view and to pinpoint interesting alterations at the molecular level. We here showed how our workflow can help in the study of a key kinase of the autophagy pathway, ULK1. We discovered that in the majority of the cases the gene expression levels are not altered or can be compensated by an up-regulation of the homologous kinase ULK2, whereas more than 30 different missense mutations altering the coding region of the gene have been identified. These mutations co-occur with mutations in ULK1 interactors fundamental for the upstream regulation of autophagy, suggesting an impairment of this process in cancer types such as uterine, stomach, skin, glioblastoma and colon cancers. Moreover, our study allowed to pinpoint that more than 50% of the mutations of ULK1 kinase domain found in the cancer samples have an effect on protein stability, which is likely to have a more pronounced effect that the residual effect on protein activity, especially if it cannot be compensated by interactions with regulators of cellular ULK1 stability, which are also altered in the samples under investigation. We identified three mutations (S184F, D102N, and A28V) that predicted with only impact on kinase activity, either altering the functional dynamics of the protein or the capability to exert long range effects from distal site to the functional and catalytic regions. Due to the paucity of experimental data on ULK1 mutations, future studies will be required to understand if these mutations have an inhibitory or activatory role on the kinase. The framework here applied could be more broadly extended to other targets of interest, as we recently started to apply, to help in the classification of mutational effects, along with prioritizing the variants for experimental validation and a specific biological readout.

## Supplementary information

Supplementary file 1

Supplementary file 2

Supplementary file 3

Supplementary file 4

Supplementary file 5

Supplementary file 6

Supplementary file 7
